# The Natural Stories corpus: a reading-time corpus of English texts containing rare syntactic constructions

**DOI:** 10.1007/s10579-020-09503-7

**Published:** 2020-09-04

**Authors:** Richard Futrell, Edward Gibson, Harry J. Tily, Idan Blank, Anastasia Vishnevetsky, Steven T. Piantadosi, Evelina Fedorenko

**Affiliations:** 1 University of California, Irvine, USA; 2 Massachusetts Institute of Technology, Cambridge , USA; 3 Viome, Inc., Seattle, USA; 4 University of California, Los Angeles, USA; 5 University of California, Berkeley, USA

**Keywords:** Cognitive modeling, Reading time, Psycholinguistics

## Abstract

It is now a common practice to compare models of human language processing by comparing how well they predict behavioral and neural measures of processing difficulty, such as reading times, on corpora of rich naturalistic linguistic materials. However, many of these corpora, which are based on naturally-occurring text, do not contain many of the low-frequency syntactic constructions that are often required to distinguish between processing theories. Here we describe a new corpus consisting of English texts edited to contain many low-frequency syntactic constructions while still sounding fluent to native speakers. The corpus is annotated with hand-corrected Penn Treebank-style parse trees and includes self-paced reading time data and aligned audio recordings. We give an overview of the content of the corpus, review recent work using the corpus, and release the data.

## Introduction

It is becoming a standard practice to evaluate theories of human language processing by comparing their ability to predict behavioral and neural reactions to fixed standardized corpora of naturalistic text. This method has been used to study several dependent variables which are believed to be indicative of human language processing difficulty, including word fixation time in eyetracking (Kennedy et al. 2013), word reaction time in self-paced reading (Roark et al. 2009; Frank et al. 2013), BOLD signal in fMRI data (Bachrach et al. 2009), and event-related potentials (Dambacher et al. 2006; Frank et al. 2015).

The more traditional approach to evaluating psycholinguistic models has been to collect psychometric measures on hand-crafted experimental stimuli designed to tease apart detailed model predictions. While this approach makes it easy to compare models on their accuracy for specific constructions and phenomena, it is hard to get a sense of how models compare on their coverage of a broad range of phenomena. Comparing model predictions over standardized texts makes it is easier to evaluate coverage.

Although the corpus approach has these advantages, the existing corpora currently used are based on naturally-occurring text, which is unlikely to include the kinds of sentences which can crucially distinguish between theories. Many of the most puzzling phenomena in psycholinguistics, and the phenomena which have been used to test models, have only been observed in extremely rare constructions, such as multiply nested object-extracted relative clauses (Roland et al. 2007). Corpora of naturally-occurring text are unlikely to contain these constructions.

Here we attempt to combine the strength of experimental approaches—which can test theories using targeted low-frequency structures—and corpus studies—which provide broad-coverage comparability between models. We introduce and release a new corpus, the **Natural Stories Corpus**, a series of English narrative texts designed to contain many low-frequency and psycholinguistically interesting syntactic constructions while still sounding fluent and coherent. The texts are annotated with hand-corrected Penn Treebank style phrase structure parses, and Universal Dependencies parses automatically generated from the phrase structure parses. We also release self-paced reading time data for all texts, and word-aligned audio recordings of the texts. We hope the corpus can form the basis for further annotation and become a standard test set for psycholinguistic models.^1^

## Related work

Here we survey datasets which are commonly used to test psycholinguistic theories, and how they relate to the current release.

The most prominent psycholinguistic corpus for English is the **Dundee Corpus** (Kennedy 2003), which contains 51,501 word tokens in 2368 sentences from British newspaper editorials, along with eyetracking data from 10 experimental participants. The full corpus is not publically available. A dependency parse of the corpus was released by Barrett et al. (2015). Like in the current work, the eyetracking data in the Dundee corpus is collected for sentences in context and so reflects influences beyond the sentence level. The corpus has seen wide use (Demberg and Keller 2008; Mitchell et al. 2010; Frank and Bod 2011; Fossum and Levy 2012; Smith and Levy 2013; van Schijndel and Schuler 2015; Luong et al. 2015).

The **Potsdam Sentence Corpus** (Kliegl et al. 2006) of German provides 1138 words in 144 sentences, with cloze probabilities and eyetracking data for each word. Like the current corpus, the Potsdam Sentence Corpus was designed to contain varied syntactic structures, rather than being gathered from naturalistic text. The corpus consists of isolated sentences which do not form a narrative, and during eyetracking data collection the sentences were presented in a random order. The corpus has been used to evaluate models of sentence processing based on dependency parsing (Boston et al. 2011, 2018) and to study effects of predictability on event-related potentials (Dambacher et al. 2006).

The **MIT Corpus** introduced in Bachrach et al. (2009) has similar aims to the current work, collecting reading time and fMRI data over sentences designed to contain varied structures. This dataset consists of four narratives with a total of 2647 tokens; it has been used to evaluate models of incremental prediction in Roark et al. (2009), Wu et al. (2010), and Luong et al. (2015).

The **UCL Corpus** (Frank et al. 2013) consists of 361 English sentences drawn from amateur novels, chosen for their ability to be understood out of context, with self-paced reading and eyetracking data. The goal of the corpus is to provide a sample of typical narrative sentences, complementary to our goal of providing a corpus with low-frequency constructions. Unlike the current corpus, the UCL Corpus consists of isolated sentences, so the psychometric data do not reflect effects beyond the sentence level.

Eyetracking corpora for other languages are also available, including the **Postdam-Allahabad Hindi Eyetracking Corpus** (Husain et al. 2015) and the **Beijing Sentence Corpus of Mandarin Chinese** (Yan et al. 2010).

## Corpus description

### Text

The Natural Stories corpus consists of 10 stories of about 1000 words each, comprising a total of 10,245 lexical word tokens in 485 sentences. The stories were developed by taking existing publicly available texts and editing them to contain many rare or marked syntactic constructions, while still retaining the same meaning, and while maintaining a high degree of overall fluency and comprehensibility as judged subjectively by the editor.^2^ The editors focused on including the following marked syntactic constructions: subject- and object-extracted relative clauses, clefts, topicalized structures, extraposed relative clauses, sentential subjects, sentential complements, local structural ambiguity (especially NP/Z ambiguity), idioms, and conjoined clauses with a variety of coherence relations. More details on these constructions are provided in Appendix. The texts and their sources are listed in Table 1. Along with the release of the texts and reading time data, we also release a document showing which marked syntactic constructions are present in which sentences.

**Table 1:** Stories with titles and sources

Story	Title	Source title	Source author
1	Boar	The Legend of the Bradford Boar^a^	E. H. Hopkinson
2	Aqua	Aqua, or the Water Baby^b^	Kate Douglas Wiggin
3	Matchstick	The Little Match-Seller^c^	Hans Christian Andersen
4	King of Birds	The King of the Birds^d^	Brothers Grimm
5	Elvis	Elvis Died at the Florida Barber College^e^	Roger Dean Kiser
6	Mr. Sticky	Mr. Sticky^f^	Mo McAuley
7	High School	Bullies	Sarah Cleaves
8	Roswell	Roswell UFO incident^g^	Wikipedia
9	Tulips	Tulip mania^h^	Wikipedia
10	Tourette’s	Tourette Syndrome Fact Sheet^i^	NINDS

^a^http://www.make4fun.com/stories/British-short-story/3917-The-Legend-of-the-Bradford-Boar-by-E-H-Hopkinsona

^b^http://fullreads.com/literature/aqua-or-the-water-baby/

^c^http://stenzel.ucdavis.edu/180/anthology/matchgirl.html

^d^http://www.apples4theteacher.com/holidays/bird-day/short-stories/the-king-of-the-birds.html

^e^http://www.eastoftheweb.com/short-stories/UBooks/ElvDie.shtml

^f^http://www.eastoftheweb.com/short-stories/UBooks/MrStic.shtml

^g^http://en.wikipedia.org/w/index.php?title=Roswell_UFO_incident&oldid=331989741

^h^http://en.wikipedia.org/w/index.php?title=Tulip_mania&oldid=329157998

^i^http://www.ninds.nih.gov/Disorders/Patient-Caregiver-Education/Fact-Sheets/Tourette-Syndrome-Fact-Sheet

**Fig. 1:**
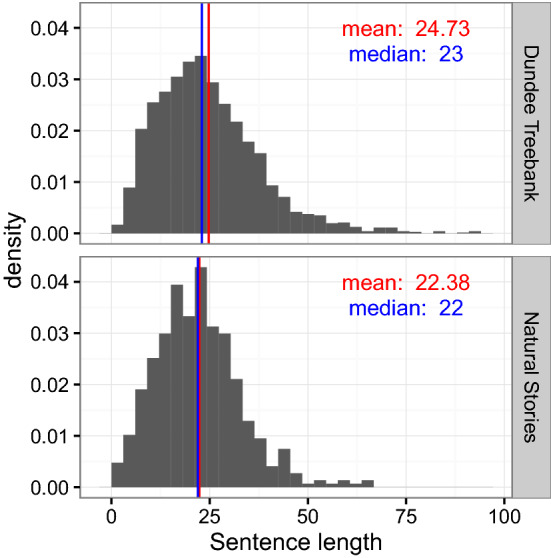
Histograms of sentence length (in tokens, including punctuation) in Natural Stories and the Dundee corpus

The mean number of tokens per sentence is 22.38, around the same as the Dundee corpus (24.73). Figure 1 shows a histogram of sentence length in Natural Stories as compared to Dundee. The word and sentence counts for each story are given in Table 2. Each token has a unique code which is referenced throughout the various annotations of the corpus, defined in the file words.tsv.

**Table 2:** Summary of stories by length

Story	# Words	# Sentences
1	1073	57
2	990	37
3	1040	55
4	1085	55
5	1013	45
6	1089	64
7	999	48
8	980	33
9	1038	48
10	938	43

Here, ‘words’ refers to lexical words

**Fig. 2:**
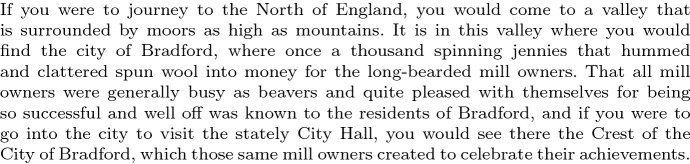
Sample text from the first story. The sample text contains marked syntactic structures: (1) an it-cleft (“It is in this valley where ...”), (2) a very long sentential subject (“That all mill owners ...was well known”), (3) an object-extracted relative clause (“..., which those same mill owners created to celebrate ...”), and others

In Fig. 2 we give a sample of text from the corpus (from the first story).

### Parses

The texts were parsed automatically using the Stanford Parser (Klein and manning 2003) and hand-corrected. Trace annotations were added by hand. We provide the resulting Penn Treebank-style phrase structure parse trees. We also provide Universal Dependencies-style parses (Nivre 2015) automatically converted from the corrected parse trees using the Stanford Parser. Deep syntactic annotations following a categorial grammar are provided by Shain et al. (2018b).

**Fig. 3:**
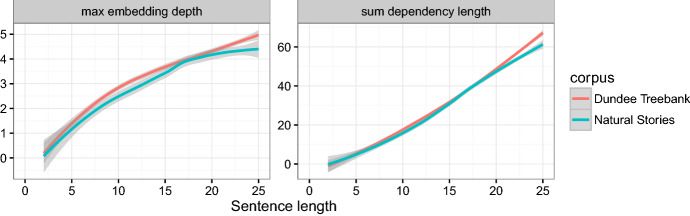
Sum dependency length and maximum embedding depth per sentence in Natural Stories (with automatic conversion to Universal Dependencies) and the Dundee Treebank (Barrett et al. 2015)

**Fig. 4:**
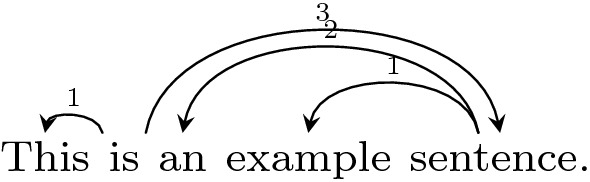
Example of sum dependency length and maximum embedding depth. Arcs are drawn from syntactic heads to syntactic dependents. Numbers over dependency arcs indicate dependency length. The sum dependency length for this sentence is 1 + 3 + 2 + 1 = 7. The maximum embedding depth = 3, because there are three arcs over the word “example”

Figure 3 shows some basic syntactic features of the corpus as compared with the Dundee corpus, computed from the dependency parses of the text. In this figure, **sum dependency length** is the total length of all dependency arcs in the dependency parse of the sentence, where length is calculated as the number of intervening words between the head and the dependent plus one, as is standard in corpus studies of dependency length (Liu 2008; Futrell et al. 2015). **Maximum embedding depth** refers to the maximum depth of a stack that would be required to parse a sentence using an incremental stack-based dependency parser such as Nivre and Scholzm (2004); it is equal to the maximum number of dependency arcs over a word at any point in a sentence. For example, the sum dependency length and maximum embedding depth of a sample sentence is calculated in Fig. 4. Although the corpus contains many low-frequency and marked constructions, its dependency length and embedding depth are not greater than the newspaper text in the Dundee corpus.

### Self-paced reading data

We collected self-paced reading (SPR) data (Just et al. 1982) for the stories from 181 native English speakers over Amazon Mechanical Turk. Text was presented in a dashed moving window display, with masked spaces. For each word, we recorded its reading time (RT) as the amount of time taken by the reader to press the button to advance to the next word. Line breaks were determined by fitting the texts to a random width of the screen, so that line breaks do not occur for the same word across participants. Each story was accompanied by 6 comprehension questions, where participants chose the correct answer from a set of two. These comprehension questions are included in our data release.

**Fig. 5:**
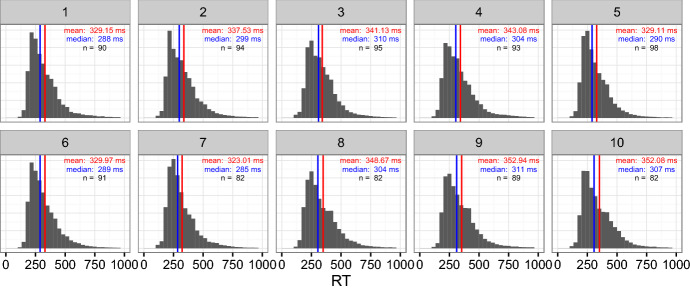
Histograms of SPR reading times (RTs) per story, after data exclusion

Each participant read 5 stories per HIT. Participants were paid $2.^3^ 19 participants read all 10 stories, and 3 participants stopped after one story. Figure 5 shows histograms of RTs per story. For this analysis, we discarded SPR data from a participant’s pass through a story if the participant got less than 5 questions correct, resulting in the exclusion of 89 passes (9% of passes excluded). We also excluded RTs less than 100 ms or greater than 3000 ms. Data exclusions of this kind and magnitude are common in psycholinguistics (see for example Boyce et al. (2020)).

#### Inter-subject correlations

**Fig. 6:**
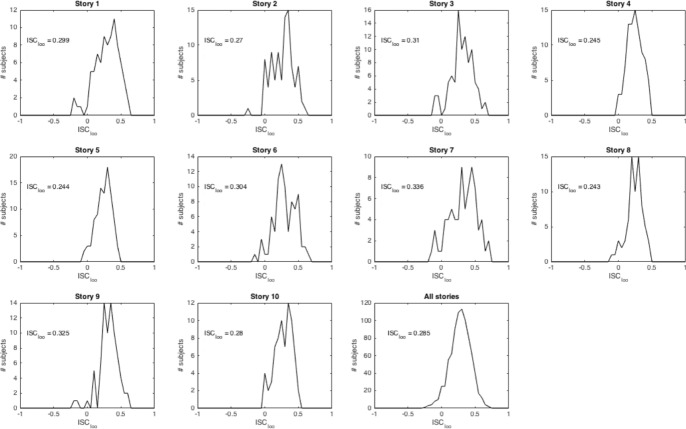
Leave-one-out inter-subject correlations (ISCs) of RTs per story. In the panels, $ISC_{loo}$ gives the average leave-one-out ISC for that story

In order to evaluate the reliability of the self-paced reading RTs and their robustness across experimental participants, we analyzed inter-subject correlations (ISCs). For each subject, we calculated the Spearman correlation of that subject’s RTs on a story with average RTs from all other subjects on that story. In this way, for each story, we get one ISC statistic per subject. Figure 6 shows histograms of these statistics per story. High correlations indicate high inter-subject reliability.

#### Psycholinguistic validation

In order to check the integrity of our RT data, we verified that it shows some of the basic effects that have been documented in the field of psycholinguistics. Some of the most robust predictors of reading time in the psycholinguistic literature are frequency, word length, and surprisal (Kliegl et al. 2004; Smith and Levy 2013). More frequent words are read more quickly, longer words are read more slowly, and more surprising words (as determined using e.g. an *n*-gram model) are read more slowly. Here we check whether these well-known effects can be found in our SPR corpus.

To do this, we fit a regression models to predict reading time based on each of the three predictors individually. For example, in order to predict the reading time from log frequency, we fit a regression of the form:

1 $$\begin{aligned} \hat{y_i} = \alpha + \beta \log f_i + \epsilon _i, \end{aligned}$$

where $y_i$ is the reading time (in ms) for the *i*th word in the corpus, $f_i$ is the frequency of the *i*th word in the corpus, and the scalars $\alpha$ and $\beta$ are chosen to minimize the sum of the squared errors $\epsilon _i$ for each word. For this analysis, we excluded outlier data and participants with low comprehension scores following the same criteria as in Sect. 3.3.

We fit a regression model as in Eq. 1 to predict reading time from log frequency, another model to predict reading time from word length (measured in orthographic characters), and another model to predict reading time from log probability under a trigram model. We expect to find a negative value of the coefficient $\beta$ when predicting reading time from frequency and trigram probability, and a positive value of the coefficient $\beta$ when predicting reading time from word length. Word and trigram counts are collected from the Google Books *n*-grams corpus, summing over years from 1990 to 2013; we make these counts available along with the corpus. Each regression is a mixed-effects regression with subject and story as random intercepts (models with random slopes did not converge), in addition to the predictors in Eq. 1. By including random intercepts, we control for by-subject and by-story variability.

The results of the regressions are shown in Table 3. In keeping with well-known effects, increased frequency and trigram probability both lead to faster reading times, and word length leads to slower reading times. These results show that basic psycholinguistic effects are present in our SPR data.

**Table 3:** Regression coefficients from individual mixed-effects regressions predicting RT for each of the three predictors log frequency, log trigram probability, and word length

Predictor	$\beta$	Std. error	*t* value
Log frequency	− 2.61	0.08	− 32.27
Log trigram probability	− 2.19	0.09	− 23.90
Word length	4.21	0.12	35.72

The first column is the predictor used in a regression predicting reading time; the column $\beta$ is the fitted regression coefficient for the predictor; Std. error is the standard error on the estimate of $\beta$, and the *t* value is the *t*-statistic for the value of $\beta$ as compared to 0. We predict and find negative values of $\beta$ for log frequency and log probability and a positive effect of word length. All *p* values are $<0.001$

### Aligned audio

We also release audio recordings of the text. These recordings are meant to be used as auditory stimuli in settings such as fMRI experiments. Five stories were read by a male (stories 1, 2, 5, 8, and 9), and the other five by a female (stories 3, 4, 6, 7, and 10).

Along with the raw audio recordings, we release time-alignments by word. The alignments were created by initial forced alignment with a proprietary text-to-audio aligner developed by Lincoln Labs, and then hand-checked and corrected by a research assistant.

### Syntactic constructions

Here we give an overview of the low-frequency or marked syntactic constructions which occur in the stories. We coded sentences in the Natural Stories corpus for presence of a number of marked constructions, and also coded 200 randomly selected sentences from the Dundee corpus for the same features. The features coded are listed by name and explained in Appendix.

**Fig. 7:**
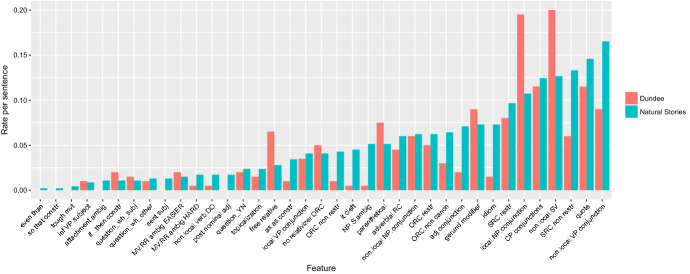
Rates of marked constructions in the Natural Stories corpus and in 200 randomly sampled sentences from the Dundee corpus

Figure 7 shows the rates of occurrence for these marked constructions per sentence in the two corpora. From the figure, we see that the Natural Stories corpus has especially high rates of relative clauses, idioms, adjective conjunction, local NP/S ambiguities, and clefts. Although there are some marked constructions which have higher frequency in Dundee than in Natural Stories, most (27/37) of the constructions are more frequent in Natural Stories than in Dundeee. Furthermore, the constructions which are especially frequent in Natural Stories are some of those which have played an important role in psycholinguistics. In particular, we point out the case of object-extracted relative clauses, which have formed the basis of much theorizing about the role of expectations and memory in human sentence processing (Grodner and Gibson 2005; Levy 2008), but which are rare in naturalistic text including Dundee (Roland et al. 2007).

## Uses of the corpus

Here we review recent work that has used the Natural Stories corpus. The corpus has primarily been used to test models of incremental difficulty in language processing: Shain et al. (2016) use it to test theories of processing slowdown due to work memory retrieval events; Shain et al. (2018a) use it to detect effects of semantic distance on language processing beyond what would be predicted by surprisal-based models; and Yan et al. (2018) use it to test whether reading times for a word in context are affected by the average diversity of contexts in which that word appears. Schijndel and Schuler (2017) and van Schijndel and Linzen (2019) have used the corpus to test explanations for a curious effect in reading times, whereby the reading time of the current word appears to be affected by the surprisal of the *following* word, and to test a neural-network model of adaptation in reading times (van Schijndel and Linzen 2018).

The corpus has also appeared in methodological studies. Shain and Schuler (2018) use the corpus to demonstrate the validity of a new methodology for controlling for “spillover” effects in self-paced reading times, where the effect of the difficulty of a word shows up in the reading times of following words.

In addition to these uses already demonstrated, the corpus has further potential applications. For example, it may be possible to use the corpus as part of a psychometric test for language comprehension, or to use the reading times from the corpus as a source of data for grammar induction models or parsers. It is our hope that, as these studies are carried out, there will be increasing interest from the computational linguistics community in reading time corpora and in psycholinguistics more generally.

## Conclusion

We have described a new psycholinguistic corpus of English, consisting of edited naturalistic text designed to contain many rare or hard-to-process constructions while still sounding fluent. We believe this corpus will provide an important part of a suite of test sets for psycholinguistic models, exposing their behavior in uncommon constructions in a way that fully naturalistic corpora cannot. We also hope that the corpus as described here forms the basis for further data collection and annotation.

## Appendix: Syntactic features coded in Sect. 3.5

Here we describe the syntactic features of the corpus which were reported in Sect. 3.5. Where necessary, we give examples of each syntactic feature. We categorize the features into conjunction features, relative clause features, ambiguity features, displacement features, and miscellaneous. We also associate each construction below with its abbreviation(s) in Fig. 7.

*Conjunction*

- **Local/nonlocal VP conjunction** (local. / non.local.)VP.conjunction. Conjunction of VPs in which the head verbs are adjacent (local) or not adjacent (nonlocal). Local example: *The man* $\underline{sang\, and\, danced}$. Nonlocal example: *The man* $\underline{sang\, a \,song\, and\, danced\, a\, dance}$.
- **Local/nonlocal NP conjunction** (local. / non.local.)NP.conjunction. Conjunction of NPs in which the head nouns are adjacent (local) or not adjacent (nonlocal). Local example: *Rewarded with* $\underline{land\, and\, fame}$. Nonlocal example: *The people of Bradford and the people who knew them*.
- **CP conjunction** CP.conjunctions. Conjunction of CPs with explicit quantifiers. Example: *I know *$\underline{that\, you\, are\, a \,doctor\, and\, that \,you\, are\, a \,criminal}$.

*Relative clauses*

- **Restrictive/nonrestrictive SRC** SRC.restr / SRC.non.restr. Subject-extracted relative clauses with either restrictive or nonrestrictive semantics. We marked relative clauses as restrictive if they served to restrict the domain of possible referents and nonrestrictive if they simply provided extra information. Restrictive example: *The man* $\underline{that\, knows \,Bob}$. Nonrestrictive example: *The snow,* $\underline{which \,was\, white}$, *fell everywhere*.
- **Restrictive/nonrestrictive ORC** ORC.restr / ORC.non.restr. Object-extracted relative clauses with either restrictive or nonrestrictive semantics. Example: *The man *$\underline{that\, Bob\, knows}$.
- **No-relativizer ORC** no.relativizer.ORC. An object-extracted relative clause without an explicit relativizer, e.g. *The man Bob knows*.
- **Noncanonical ORC** ORC.non.canon. An object-extracted relative clause where the subject of the relative clause is not a pronoun. Example: *The man that the woman knows*.
- **Adverbial relative clause** adverbial.RC. An relative clause with an extracted adverbial. Example: *the valley *$\underline{where \,you \,would\, find \,the\, city\, of\, Bradford}$.
- **Free relative clause** free.relative. A relative clause not modifying a noun. Example: $\underline{What \,I \,know}$*is that Bob is a doctor.*

*Ambiguity*

- **NP/S ambiguity** NP.S.ambig. A local ambiguity where it is unclear momentarily whether a clause is an NP or the subject of a sentence. For example, in the sentence *I know* ${\underline{Bob}}$* is a doctor*, after reading *I know Bob* it is not clear whether Bob is an NP object of *know* or the beginning of an embedded clause.
- **Main verb/reduced relative ambiguity (easy/hard)** MV.RR.ambig.EASIER / MV.RR.ambig.HARD. A local ambiguity between a main verb and a reduced relative clause. For example, *The horse* ${\underline{raced}}$ *past the barn fell*. We divide these into easy and hard cases based on the annotators’ judgment about how confusing the local ambiguity is in context.
- **PP attachment ambiguity** attachment.ambig. A global ambiguity where a PP could attach to one of two NPs. For example, in a sentence such as *The daughter of the colonel on the balcony*, it is not clear whether it is the daughter or the colonel who is on the balcony.

*Displacement*

- ***Tough*** **movement** tough.mvt. Cases where an adjective is modified by an infinitive verb phrase from which an object has been extracted. Example: *The point is* $\underline{hard\, to\, see}$.
- **Parentheticals** parenthetical. Additional material that interrupts or lies outside the syntactic structure of the rest of the sentence; constructions that would be marked as “parataxis” in Universal Dependencies. These do not necessarily have to be marked with orthographic parentheses. Example: *There was once,* $\underline{legend\, has\, it}$, *a fearful boar*.
- **Topicalization** topicalization. Cases where an NP is moved to the front of a sentence to serve as its topic. Example: $\underline{The \,history \,of\, Korea}$,* I know nothing about*.
- **Question with** *wh* **subject** question_wh_subj. Questions with *wh*-movement of the subject. Example: *Who walked into the room?*
- **Question with other ***wh* **word** question_wh_other. Questions with *wh*-movement of anything other than the subject. Example: *Who did Bob see?*

*Miscellaneous*

- **Nonlocal SV** non.local.SV. The appearance of any material between a verb and the head of its subject. Example: *The man with the hat ran away.*
- **Nonlocal verb/DO** non.local.verb.DO. The appearance of any material between a verb and its direct object. Example: *The man ate quickly the sandwich.*
- **Gerund modifiers** gerund.modifier. A case of a verb phrase modifying a noun. Example: *The man* $\underline{walking \,down \,the \,street}$ *is tall*.
- **Sentential subject** sent.subj. A sentence where the subject is an embedded clase. Example: $\underline{The\, fact\, that\, Bob\, is\, a\, doctor}$* is interesting*.
- **Infinite VP subject** inf.VP.subject. A sentence whose subject is an infinite verb phrase. Example: $\underline{To\, eat \,sweets}$ *is forbidden*.
- **Postnominal adjectives** post.nominal.adj. Adjectives which follow their nouns. Example: *The moon,* $\underline{full \,and \,bright}$.
- **Idiom** idiom. Any idiomatic expression, such as *busy as beavers*.
- **Quotation** quotation. Any directly-reported speech. Example: *The woman said*$\underline{``I\, am \,here''}$.
- **It-clefts** it.cleft. Example: $\underline{It\, was\, Mary}$* that Bob saw*.
- **even...than construction** even.than. Example: *Even taller than Mary.*
- **if...then construction** if...then.constr. Example: *If you go, then I go.*
- **as...as construction** as.as.constr. Example: *Bob was as angry as Mary.*
- **so...that construction** so.that.constr. Example: *Bob was so angry that he was shaking.*
- **Yes-no Question** question_YN. Example: *Is Mary here?*

## Footnotes

1 The corpus is available from http://github.com/languageMIT/naturalstories. This corpus is distributed under an Attribution-NonCommercial-ShareAlike (CC BY-NC-SA) license, allowing free modification and re-distribution of the corpus so long as derivative work is released under the same terms.

2 The editors were Anastasia Vishnevetsky, Edward Gibson, Evelina Fedorenko, and Steven T. Piantadosi.

3 The SPR experiment was carried out in early 2011, when such wages were common on Mechanical Turk.
